# SmacN7 enhances the sensitivity of pancreatic cancer cells to tumor necrosis factor-related apoptosis-inducing ligand or gemcitabine

**DOI:** 10.3892/ol.2013.1285

**Published:** 2013-04-03

**Authors:** WUYUAN ZHOU, JUN SHI, ZUOXING NIU, HONGMIN LUO, HE TIAN, JIE GAO, FACHANG YU, SHENG LI

**Affiliations:** 1 Department of Surgery, Shandong Cancer Hospital, Jinan 250017;; 2 Blood Center of Shandong, Jinan 250014;; 3 Department of Hepatobiliary Surgery, Shandong Cancer Hospital, Jinan 250017;; 4 Key Laboratory for Rare and Uncommon Diseases of Shandong, Shandong Academy of Medical Sciences, Jinan 250017;; 5 Key Laboratory for Rare and Uncommon Diseases of Shandong, Shandong Academy of Medical Sciences, Jinan 250012, P.R. China

**Keywords:** pancreatic cancer, Smac-mimic polypeptide, Smac/DIABLO, tumor necrosis factor-related apoptosis-inducing ligand, gemcitabine, apoptosis

## Abstract

The aim of this study was to investigate the effect of SmacN7 on the biological characteristics of pancreatic cancer cell lines, and to assess the effect of SmacN7 on the sensitivity to tumor necrosis factor (TNF)-related apoptosis-inducing ligand (TRAIL) and gemcitabine. SmacN7 fusion polypeptide was synthesized and characterized using mass spectrometry. The morphology of apoptotic SW1990 cells and apoptotic rates were observed after 24 h of SmacN7 treatment, and the changes of cell growth inhibition rate were investigated following treatment with different concentrations of SmacN7. The combined effects of SmacN7 and different concentrations of TRAIL or gemcitabine for 24 h on the apoptotic rates of SW1990 cells were assessed, and the changes of expression of apoptosis-related proteins including X-linked inhibitor of apoptosis protein (XIAP), cytochrome C and caspase-3 were determined. Mass spectrometric identification of SmacN7 was fully consistent with the expected results. The cell growth inhibition rates of SW1990 cells 24 h post-treatment with TRAIL at different concentrations were 18.11, 37.67, 42.63 and 67.6%, in comparison to 17.65, 31.85, 40.11 and 74.99% following combined treatment of SmacN7 and different concentrations of gemcitabine for 24 h. The combined treatment of SmacN7 and gemcitabine for 24 h resulted in significantly elevated expression of cytochrome C and caspase-3 cleavage fragment, p17, and a significant reduction in XIAP expression (P<0.05). SmacN7 inhibits pancreatic cell growth. The inhibition rates of SW1990 cells caused by treatment with various concentrations of SmacN7 appear in a time- and concentration-dependent manner. The TRAIL- or gemcitabine-induced apoptosis of pancreatic cancer cells, enhanced by SmacN7, may be associated with the activity of intracellular pro-apoptotic proteins such as Smac/DIABLO (second mitochondria-derived activator of caspase/direct IAP binding protein with low PI), cytochrome C, XIAP and caspase-3.

## Introduction

Tumor necrosis factor (TNF)-related apoptosis-inducing ligand (TRAIL), first identified and cloned by Wiley *et al*(1) in 1995, is a member of the TNF family, and functions by binding to death receptors and activating the death receptor apoptosis pathway. The TRAIL gene is located on chromosome 3 at position 3q26, which consists of 281 amino acids and belongs to a type II membrane protein (glycoprotein). Its C-terminal extracellular domain shows clear homology to other TNF family members.

Cumulative evidence from studies demonstrates that multiple tumor cells are tolerant to TRAIL-induced apoptosis, and that repeated use of TRAIL may lead to the emergence of resistance to TRAIL in some sensitive cancer cells (2–5), limiting the application of TRAIL in the treatment of many tumors. Reduced expression or mutation of TRAIL death receptor DR4 (6,7), dysfunction of caspase-8 (4), lack of Smac/DIABLO in the cytoplasm or a reduction in the release of Smac/DIABLO from mitochondria (8), overexpression of Bcl-2, loss of Bax or Bak function (3), or overexpression of the inhibitor of apoptosis (IAP) family of proteins, including X-linked inhibitor of apoptosis protein (XIAP), may all lead to the development of resistance of tumor cells to TRAIL.

Mitochondrial pro-apoptotic protein, as a type of IAP protein, promotes apoptosis upon stimulation with an apoptotic signal. The N-terminal Ala-Val-Pro-Ile (AVPI) sequence forms an important domain to bind to IAPs and exhibit apoptosis, in which the Ala residue inserts into a pocket of the surface groove on BIR3, and binds to BIR3 via a hydrogen bond. Smac, on the other hand, only functions in cytoplasm (9). The AVPI peptide of natural Smac cannot effectively penetrate the cell membrane, and suffers from problems of instability, easy degradation, low utilization rate and inadequate affinity with XIAP. The antennal transcription factor of *Drosophila melanogaster* is an important membrane-regulating protein, in which the protein transduction domain, consisting of RQIKIWFQNRRMKWKK, has transmembrane transport ability, which may guide the entry of polypeptides and proteins into target cells (10).

The present study targeted the AVPI of the protein Smac. AVPIAQK peptide was synthesized using the Fmoc chemistry method (solid-phase peptide synthesis), and was then bound to the protein transduction domain of the antennal transcription factor of *Drosophila melanogaster,* with cell membrane penetration via proline to synthesize the Smac-mimic proapoptotic polypeptide SmacN7. The effect of exogenous Smac-mimic polypeptide on the apoptosis of SW1990 cells was assessed at different times following treatment at various concentrations. In addition, the relationship between the TRAIL-activated death receptor apoptosis pathway and the Smac/DIABLO-induced mitochondrial pathway was investigated.

## Materials and methods

### 

#### Design, synthesis and purification of Smac-mimic polypeptide SmacN7

Based on the four AVPI residues on Smac N-terminus, an AVPIAQK peptide sequence with a similar KD value to that of the binding of mature Smac AVPI to XIAP-BIR3 was selected as the synthesis sequence, whilst the antennal factor sequence RQIKIWFQNRRMKWKK (10) with cell membrane penetration served as a vector peptide (AC Scientific, Inc., Xi’an, China). These were connected via proline. The synthesis was performed using the standard artificial solid-phase synthesis protocol on a solid-phase peptide synthesizer. Single amino acids were sequentially added into the C-terminus of AVPI and the terminal was labeled with biotin. Resin was used for support of the solid-phase synthesis, and the flow phase contained 0.1% trifluoroacetic acid (TFA; Tedia, Pudong, Shanghai, China) aqueous solution/0.1% trifluoroacetic acid-CH3CN aqueous solution. The target polypeptide was synthesized through multiple-step reactions. The synthesized peptide was purified using a high-performance liquid chromatography system (Waters 600E; Ledon Technologies, Suzhou, Jiangsu, China) with a column of C18 (20×50 mm). When the purity was more than 95%, a rotary evaporator was used for vacuum pumping. After being frozen at a low temperature and dried, the crystalline solid was collected, weighed using an electronic balance, packaged separately at 1 mg per bottle, and stored at −20°C for the subsequent experiment.

#### Plotting of cell growth curve

The grown cells that were almost fused were harvested, digested with pancreatin, prepared into suspensions using new medium and counted. Cells were then transferred into 21 bottles for passage at a concentration of 5×10^4^/ml per bottle. After 24 h, the cells were counted, and then counted again, once every 24 h. The number of cells in 3 bottles was counted, respectively, and the mean number was calculated. The cells were counted for 7 successive days in total. Based on the cell counts, the cell growth curve was plotted based on the cell number per unit (cells/ml, vertical coordinate) and time (horizontal coordinate).

#### Drug preparation

The Smac-mimic polypeptide SmacN7 was formulated into a storage solution at a concentration of 500 *μ*g/ml with phosphate-buffered saline (PBS); TRAIL (Chemicon, Temecula, CA, USA) was formulated into a storage solution of 5 *μ*g/ml with PBS; gemcitabine (Lilly France S.A., Lilly, Fegersheim, France) was formulated into a storage solution of 60 *μ*mol/l using RPMI-1640 medium, which was then stored at −20°C, and the working concentration was prepared using RPMI-1640 medium. The TRAIL was then diluted into concentrations of 200, 500, 1,000 and 2,500 ng/ml, and gemcitabine was prepared into 10, 20, 40 and 60 mol/l for the subsequent experiment.

#### Apoptotic rate detected by flow cytometry (FCM)

SW1990 cells (Shandong Cancer Institute, Shandong Province Key Laboratory of Radiation Oncology), at a concentration of 5×10^6^/ml, were seeded in a 25-ml culture bottle. SW1990 cells at a concentration of 5×10^6^/ml, were seeded in a 25-ml culture flask. Then the cells were repeatedly cultured in five flasks. Cells were placed in an incubator at 37°C containing 5% CO_2_ for 24 h. When the majority of the cells were adherent to the wall of the bottle, the cells were cultured at 4°C for a further 2 h to promote synchronous cell growth. Some cells were harvested to prepare single-cell suspensions, and then treated with the Smac-mimic polypeptide SmacN7 at a concentration of 500 *μ*g/ml. After 24 h of treatment, the cells were harvested, transferred into a 5-ml tube, centrifuged at 1,500 × g for 15 min and then washed in PBS twice. Cells were then re-suspended in 0.5 ml of pre-cooled 1X binding buffer, supplemented with 1.25 *μ*l of Annexin V-FITC (200 *μ*g/ml; Alexis Biochemicals, San Diego, CA, USA), incubated in the dark, at room temperature for 15 min and centrifuged at 1,000 × g at room temperature for 5 min to remove the supernatant. Cells were then re-suspended in 0.5 ml of pre-cooled 1X binding buffer, supplemented with 10 *μ*l of propidium iodide (PI), and detected on a flow cytometer. In the two-parameter scatter plot diagram of FCM, the Annexin V^−^/PI cells in the left lower quadrant served as controls, the Annexin V^+^/PI^−^ cells in the right lower quadrant were defined as apoptotic cells, and the Annexin V^+^/PI^+^ cells in the right upper quadrant were defined as necrotic cells. The apoptotic rate was then calculated.

#### Apoptosis detected by Hoechst 33342 staining

The pancreatic cancer SW1990 cells were seeded on 12-well plates, and cultured routinely until ∼80% were fused. Physiological saline and 500 *μ*g/ml of SmacN7 were added, and, after a further culture of 24 h, Hoechst 33342 staining solution (Invitrogen Life Technologies, Carlsbad, CA, USA) was added to yield a concentration of 10 *μ*g/ml. Cells were then cultured in the dark at 37°C for 15–20 min. Under a fluorescence microscope, the ultraviolet of the Hoechst 33342, excited by a Krypton laser, produced a blue fluorescence. Although the normal cells and the middle- and early-stage apoptotic cells were all stained by Hoechst 33342, the nuclei of the normal cells stained with Hoechst 33342 appeared round and light blue, whereas the nuclei of the apoptotic cells appeared bright blue, with a debris-like shape.

### MTT assay detects inhibition of SmacN7 on growth of pancreatic cancer cell lines

Cell culture. The pancreatic cancer cells were cultured routinely in 25-ml bottles, and, when 80% of the cells were fused, were digested using 1 ml of 0.25% trypsin. When microscopy revealed that the cells had turned round and that some had dropped from the bottle wall, 4 ml of medium-containing serum was added to terminate the digestion. The cells were then prepared into a single-cell suspension in the culture bottle. The cell concentration and number were counted using the blood count plate under a microscope. The single-cell suspension was diluted into solution of 1×10^5^ cells/l with the medium. The pancreatic cancer cells were seeded on 96-well plates, and 100 *μ*l of the aforementioned single-cell suspension was added to each well, with the exception of the blank control well. The pancreatic cancer cells seeded onto the 96-well plates were routinely cultured until 80% of fusion.

#### SmacN7 treatment and MTT assay

The SW1990 cells were seeded onto 96-well plates, supplemented with 50, 100, 200 and 500 *μ*g/ml of SmacN7 and cultured for 4 h. Mitomycin C (0.05 mg/ml) was then added, leaving 200 *μ*l of total reaction system in each well. Five replicate wells were set for each concentration, and the cells without drug treatment served as controls. Following a further culture for 24, 48 and 72 h, 20 *μ*l of MTT solution (5 mg/ml, formulated with PBS) was added to each well, and the cells were cultured for a further 4 h. The supernatant was then removed, and 200 *μ*l of dimethyl sulfoxide (DMSO) was added to each well, prior to 10 min oscillation. The absorption values *A*1 and *A*2 on 550 nm and 630 nm of each well were then measured using the microplate reader. The *A* value of each well was defined as *A*1-*A*2, and in the current study, the mean of the *A* values in 5 replicate wells was described as the *A* value of the well. The cell growth inhibition rate (CGIR) was calculated using the following formula. CGIR (%)=(1−*A* value in the experiment group)/A value ×100. All experiments were repeated 3 times, and the mean value was calculated.

#### Combined treatment of SmacN7 and different concentrations of TRAIL and MTT assay

Cells were cultured as described above. The inhibition of 500 *μ*g/ml of SmacN7 in combination with TRAIL at concentrations of 200, 500, 1,000 and 2,500 ng/ml on the growth of SW1990 cells was assessed.

#### Combined treatment of SmacN7 and different concentrations of gemcitabine and MTT assay

Cells were cultured as described above. The inhibition of 500 *μ*g/ml of SmacN7 in combination with gemcitabine at concentrations of 10, 20, 40 and 60 *μ*mol/l on the growth of SW1990 cells was assessed.

#### Western blot determines expression of XIAP, cytochrome C and caspase-3 proteins

After 24 h of combined treatment of SmacN7, TRAIL (500 ng/ml) and gemcitabine (20 *μ*mol/l), the SW1990 cells were digested with 0.25% pancreatin. The cell sedimentation was harvested, combined with 400 *μ*l of lysis buffer, centrifuged, and then sodium dodecyl sulfate-polyacrylamide gel electrophoresis (SDS-PAGE) was performed. The electrophoresed product was transferred onto a nitrocellulose (NC) membrane, which was then placed into the buffer containing DAB at room temperature. When the protein color reached the reaction requirement, the membrane was transferred to another dish to cease the reaction. The protein expression was analyzed using the digital gel imaging system.

#### Statistical analysis

All data were expressed as mean ± standard deviation (SD), and all statistical analyses were performed using the statistical software SPSS version 11.5. The difference between the means of two samples was tested for statistical significance using a t-test, while one-way analysis of variance in combination with the least significant difference (LSD) was used to compare multiple samples. P<0.05 was considered to indicate a statistically significant result.

## Results

### 

#### Purification and mass spectrometric identification of SmacN7

SmacN7 is a penetrating fusion polypeptide synthesized by seven residues AVPIAQK and RQIKIWFQNRRMKWKK on the protein transduction domain of the antennal transcription factor of *Drosophila melanogaster*, with a molecular weight of 3278.08. Following solid-phase polypeptide purification and reverse-phase HPLC purification, the purity of the peak of the product reached more than 95%. Mass spectrometric identification demonstrated that the molecular ion peak of SmacN7 was fully consistent with the expected result.

#### Effect of SmacN7 on morphology and growth of SW1990 cells

**Figure 1:**
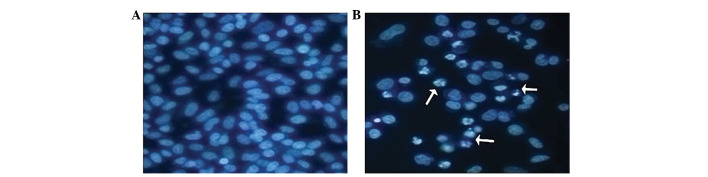
The morphology of apoptotic SW1990 cells 24 h after treatment with SmacN7. (A) the SW1990 cells without treatment; (B) the SW1990 cells treated with SmacN7 (magnification, ×400).

Following treatment with SmacN7 and Hoechst 33342 staining, SW1990 cells showed enlarged cell bodies and nuclei, mild intracellular swelling and a short-shuttle shape appearance. FCM detected cell cycle arrest during the G0/G1 phase, reduction in cell percentage at S phase and slow cell growth (Fig. 1).

#### Inhibition of SmacN7 on growth of SW1990 cells

The CGIRs of SW1990 cells caused by treatment of SmacN7 at concentrations of 50, 100, 200 and 500 *μ*g/ml for 24, 48 and 72 h were determined by MTT assay, and the results showed that the CGIRs appeared in a time- and concentration-dependent manner (P<0.05) (Table I).

#### Combined treatment of SmacN7 and various concentrations of TRAIL or gemcitabine inhibit growth of SW1990 cells

Combined treatment of SmacN7 at a concentration of 500 *μ*g/ml and TRAIL at concentrations of 200, 500, 1,000 and 2,500 ng/ml for 24 h achieved CGIRs of 318.11%, 37.67%, 42.63% and 67.6% respectively, in comparison to 17.65%, 31.85%, 40.11%, 74.99% following treatment with SmacN7 in combination with gemcitabine at concentrations of 10, 20, 40 and 60 *μ*mol/l respectively.

#### Combined treatment of SmacN7, TRAIL and gemcitabine on expression of apoptosis-related proteins in SW1990 cells

**Figure 2:**
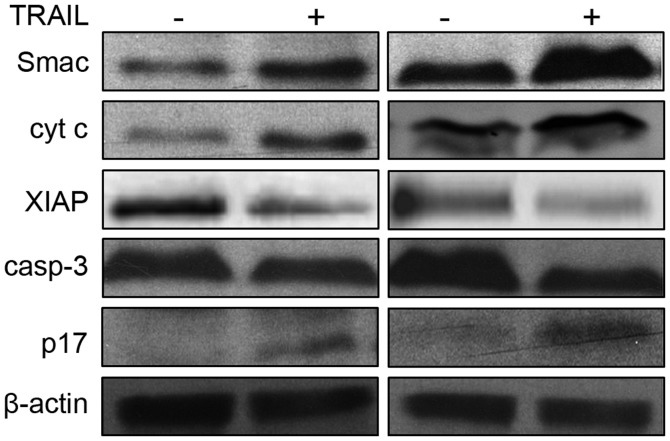
Expression of apoptosis-related proteins in SW1990 cells 24 h after treatment with TRAIL at a concentration of 500 ng/ml. TRAIL, tumor necrosis factor-related apoptosis-inducing ligand; XIAP, X-linked inhibitor of apoptosis protein.

Combined treatment of SmacN7 and TRAIL (500 ng/ml) for 24 h led to elevated expression of Smac/DIABLO, cytochrome C, and caspase-3 cleavage fragment, p17, and a reduction in XIAP protein expression. However, the changes in protein expression in TRAIL-treated SW1990 cells were greater than those in the control cells (Fig. 2). Following combined treatment of SmacN7 and gemcitabine (20 *μ*mol/l) for 24 h, the expression of Smac/DIABLO, cytochrome C and p17 was elevated, while the XIAP protein expression was reduced, which was similar to those post-treatment with TRAIL (500 ng/ml). In addition, the changes in protein expression in TRAIL-treated SW1990 cells were greater than those in the control cells.

## Discussion

Currently, it is widely recognized that there are two pathways of apoptosis (11–13): the death receptor pathway and the mitochondrial pathway. In the death receptor apoptosis pathway, death ligand-like TRAIL binds to the death receptor (mainly TNF family receptors including TNFR1, TNFR2, Fas, DR4 and DR5) on the cell surface, then the death domain in the intracellular fragment of the death receptor attracts an adaptor protein such as Fas-associated protein with death domain (FADD) or tumor necrosis factor receptor type 1-associated death domain protein (TRADD), and recruits caspase-8 and caspase-10 precursors. This leads to the formation of a death-induced signal complex (DISC) and the production of active initiator caspases, followed by the activation of effector caspases such as caspase-3, caspase-6 and caspase-7, thereby inducing apoptosis. In the mitochondrial apoptosis pathway, many apoptotic signals, such as DNA damage, external stimuli and some chemotherapeutic drugs, can induce the release of cytochrome C and Smac/DIABLO from mitochondria into the cytoplasm. Cytochrome C binds to apoptotic protease-activating factor (Apaf-1), recruits caspase-9 precursor to form an apoptosome, produces active caspase-9, and then activates downstream effector caspases to induce apoptosis.

Smac/DIABLO does not induce apoptosis in normal cells, but only functions in damaged cells (14). Under normal circumstances, Smac/DIABLO is predominantly present in the mitochondria, but is released from the mitochondria into the cytoplasm when cells undergo apoptotic stimuli such as anti-cancer drugs, or chemical or physical apoptotic signals. These apoptotic stimuli promote apoptosis by interacting with IAPs, thus leading to a relief of the IAP-mediated inhibition of caspases (14,15).

Smac/DIABLO can disrupt the interaction between XIAP-BIR3 and caspase-9, and Linker-BIR2 and caspase-3 or caspase-7, leading to a relief of the XIAP-mediated caspase inhibition. The four N-terminal AVPI residues (Smac-4) of Smac/DIABLO play a critical role in Smac/DIABLO functions. Numerous studies have demonstrated that the AVPI sequence is capable of binding to the BIR domain, which is the functional basis of Smac/DIABLO (16). It has been demonstrated that the four N-terminal residues of caspase-9 linker peptide share significant homology with the N-terminal tetra-peptide in mature Smac. Therefore, removal of the inhibition of caspase-9 by XIAP promotes activation of caspase-9 and the subsequent activation and apoptosis of caspase-3.

There is competition between Smac/DIABLO and IAP. Therefore, an increase in Smac/DIABLO concentration leads to the promotion of apoptosis. In addition, Smac/DIABLO may regulate IAP functions through other mechanisms. It has been indicated that Smac/DIABLO increases ubiquitin ligase activity of IAP, which degrades IAP proteins, leading to a reduced amount of IAP. If Smac/DIABLO can selectively lead to ubiquitination of c-IAP1 and c-IAP2, resulting in reduction in concentrations of both proteins (17), a further increase in relative concentration of Smac/DIABLO may promote apoptosis. In addition, it has been demonstrated that the co-presence of Smac/DIABLO and other molecules reduces degradation through the inhibition of Smac ubiquitination, for example, the interaction between NADE, previously known as p75NTR-associated cell death executor, and Smac/DIABLO decreases Smac ubiquitination (18), which is beneficial for Smac/DIABLO functioning. Based on the TRAIL-regulated apoptosis pathway, it is considered that Smac/DIABLO plays a critical role in TRAIL-induced apoptosis (19). The present study investigated the effect of SmacN7 on cell growth and TRAIL sensitivity, and found that exogenous Smac-mimic polypeptide affected the growth of pancreatic cancer SW1990 cells. The inhibition of treatment with SmacN7 at various concentrations on SW1990 cells appeared in a time- and concentration-dependent manner. The TRAIL- or gemcitabine-induced apoptosis of pancreatic cancer cells, enhanced by Smac-mimic polypeptide, may be associated with the activity of intracellular pro-apoptotic proteins such as Smac/DIABLO, cytochrome C, XIAP and caspase-3.

## Figures and Tables

**Table I:** Inhibition of SW1990 cell growth 24–72 h after treatment with different concentrations of SmacN7.

	Concentration of SmacN7 (*μ*g/ml)			

Time following treatment (h)	50	100	200	500
24	18.11±0.96	26.03±1.33	37.08±1.26	46.19±1.41
48	20.23±1.11	38.35±1.13	48.96±1.14	72.55±1.38
72	22.64±0.80	48.01±0.92	61.82±1.33	77.18±1.17
